# Phytochemical Analysis and Investigation of the Antimicrobial and Cytotoxic Activities of *Croton dichogamus* Pax Crude Root Extracts

**DOI:** 10.1155/2021/2699269

**Published:** 2021-07-23

**Authors:** Dorine Nyak Matara, Joseph Mwanzia Nguta, Fredrick Mutie Musila, Isaac Mapenay

**Affiliations:** ^1^Department of Public Health, Pharmacology and Toxicology, Faculty of Veterinary Medicine, University of Nairobi, Nairobi, Kenya; ^2^Department of Applied and Technical Biology, School of Biological and Life Sciences, Technical University of Kenya, Nairobi, Kenya

## Abstract

**Background:**

Increasing antimicrobial resistance has led to an arduous search for new potent drugs from nature. In this search, plants have proved to be rich reservoirs of efficacious medicinal components that manage ailments. The current study is designed to investigate the phytochemical composition, antimicrobial activity, and the cytotoxicity of the crude root extracts of *Croton dichogamus*, a shrub that is commonly used in the eastern Africa for the management of infectious diseases.

**Methods:**

The roots of *Croton dichogamus* were obtained, dried, ground, and extracted using three solvents (acetone, distilled water, and 50% ethanol). The antimicrobial activity was tested using agar well diffusion and microbroth dilution techniques against five human pathogens. The brine shrimp lethality assay was used to assess the toxic effect.

**Results:**

The phytochemical screening indicated the presence of terpenoids, flavonoids, tannins, phenols, polyuronides, saponins, and anthracenes. The brine shrimp lethality assay indicated that all the extracts were highly cytotoxic with LC_50_ values below 100 *μ*g/ml. Acetonic extract had an LC_50_ value of 4.148 *μ*g/ml, hydroethanolic extract had 76.09 *μ*g/ml, and aqueous extract had 42.61 *μ*g/ml. All extracts showed the antibacterial activity against Gram-positive bacteria (*B. cereus* and *S. aureus*) and a fungal organism, *C. albicans*. The extracts showed no antibacterial effect on the Gram-negative bacterial strains (*P. aeruginosa* and *E. coli*) at a concentration of 250 mg/ml. The highest antimicrobial activity was demonstrated by the acetonic extract on *B. cereus* which had an MIC of 10.42 mg/ml and a zone of inhibition of 17.33 ± 0.58 at a concentration of 250 mg/ml.

**Conclusion:**

In this research work, we report that *C. dichogamus* had the antimicrobial activity confirming the folklore claim. The results made a strong case for isolation of novel anticancer lead compounds.

## 1. Introduction

The emergence and propagation of antimicrobial resisting strains of microbes in clinical practice have extremely reduced the efficacy of antimicrobial weaponry, resulting to recurrence of therapeutic failure and mortality cases [1, 2]. Therefore, there is an urgent need for new effective antimicrobial agents [3]. Since classical times, human beings have been in a ceaseless search for ways of relieving such diseases, leading to great discoveries on the use of natural ways (like plants) of treating complex ailments [4]. Over the last three decades, the use of traditional medicine has immensely grown with approximately 80% of worldwide population relying on this system as the chief form of treatment [5]. The use of medicinal plants for reliving common ailments has been accepted widely because of their accessibility, availability, effectiveness, and affordability [6]. Various studies have demonstrated that antimicrobials of plant origin possess a huge potential to fight against bacterial, protozoal, viral, and fungal diseases with minimum complications [7]. The screening performed on numerous plant extracts and their natural products has also shown that vegetative greeneries and their secondary metabolites have a notable activity against a wide range of microbes when used alone or they could also act as synergists or potentiators of other antimicrobial agents [8]. The World Health Organization (WHO) also acknowledges that traditional medicine is a prime healthcare system that gives good results to its users [9].

The plants that have been used extensively in traditional medicine are those of *Croton* genus (Euphorbiaceae family) that has 1300 species. Most of these species have displayed remarkable ability to manage a broad spectrum of diseases [6, 7]. *Croton dichogamus,* one of its species, is a medicinal shrub growing in Kenya, Tanzania, Somalia, Rwanda, Ethiopia, Mozambique, and Madagascar, where it often plays a significant role in traditional medicine [10].In Tanzania, the powdered roots of “Mhande,” *C. dichogamus* are used by Sukuma to treat tuberculosis, while the smoked roots are used to relive chest pains and fevers [5]. In the Kenyan Lake Basin, among the Luo community, *C. dichogamus* (Rachar) is used to manage respiratory diseases such as asthma, pneumonia, and cough [9–11]. It is used in Somalia to treat gonorrhea [12], arthritis [5], stomach, and back pains [11, 12]. The Mbeere and Afan Oromo communities in Tanzania use dried leaves, roots, and stem barks of *C. dichogamus* as an antimalarial, antipyretic [5], and as a pesticide [13]. In Kitui County, the Kamba community prepares an infusion of the stem bark and leaves of *C. dichogamus,* locally known as “Mwalula,” for drinking to alleviate back pains, malaria, stomachache, chest problems, fever, oedema, and cough [14]. In the same community, a root decoction is also drunk for the treatment of impotence and infertility [14].

Previous reports have indicated that ethanolic extract of *C. dichogamus* had an antibacterial activity against two *Mycobacterium* species, namely, *Mycobacterium indicus pranii* and *Mycobacterium madagascariense indicus,* giving a minimum inhibitory concentration (MIC) value of 1.25 mg/ml [15]. Studies have also shown that the essential oils from leaves of *C. dichogamus* had an antimalarial activity against *Anopheles gambiae* [16]. The antiproliferative activity [17], insecticidal activity [13], and hypocholesteremic activity [18] have also been reported.

Despite the extensive use of *C. dichogamus* for curative purposes and the traditional claim of its efficacy in the management of common ailments, the available literature is scanty. The pharmacological activity, antimicrobial property, and safety of *C. dichogamus* have never been investigated and documented. The motive of the current study was to evaluate the antimicrobial activity of acetonic, hydroethanolic, and aqueous root extracts of *C. dichogamus* against five human pathogens (*Staphylococcus aureus*, *Bacillus cereus*, *Pseudomonas aeruginosa*, *Escherichia coli*, and *Candida albicans*) using agar well diffusion and microbroth dilution techniques. The phytochemical composition was evaluated, and the safety of the extracts was determined in a bench top assay using brine shrimp larvae (*Artemia salina*) [19].

## 2. Materials and Methods

### 2.1. Chemicals, Reagents, and Drugs

Absolute ethanol (Loba Chemic Pvt., India), acetone (Nice Chemicals Pvt., Ltd., India), dimethyl sulfoxide (Loba Chemic. Pvt., India), brine shrimp eggs (A.A Biotech Pvt., India), vincristine sulphate (Celon Laboratories Pvt., India), Mueller Hinton agar (HI-Media Laboratories Pvt., India), Mueller Hinton broth (HI-Media Laboratories Pvt., India), cephalexin (Medisel Ltd., Kenya), and fluconazole (Dawa Ltd., Kenya) were used in this study, and the chemical and reagents were of analytical grade.

### 2.2. Plant Collection and Authentication

Fresh roots of the shrub *C. dichogamus* were collected from Kisumu East Subcounty (0° 14′ 60.00″ N, 34° 54′ 59.99″ E), Nyanza province, in the month of November 2020 and transported to the Department of Public Health, Pharmacology and Toxicology, University of Nairobi. The taxonomic identification was performed by Mr. Ken Matheka at the East African Herbarium located at the National Museum of Kenya. A voucher specimen of reference number of NMK/BOT/CTX.1/2 was deposited at the East African Herbarium.

### 2.3. Extraction of Plant Material

The fresh roots of *C. dichogamus* were washed, chopped into small pieces, air-dried, and ground into fine powder. The resulting plant powder was packed in sterile airtight ziplock bags and stored in a cool, dry shelf awaiting extraction.

Aqueous extract was prepared by cold maceration by adding 2000 ml of distilled water to 500 gm of the root powder. The mixture was then macerated for 72 hours with vigorous shaking in the morning and evening and then filtered. The filtrate was kept in a deep freezer for 24 h and then lyophilized to form a light brown powder which was stored in an amber bottle at −4°C in a refrigerator.

Hydroethanolic (50% ethanol) extraction was performed by taking 500 gm of the *C. dichogamus* root powder into an extraction jar and then adding 1000 ml of distilled water followed by 1000 ml of absolute ethanol. The mixture was macerated for 72 hours with vigorous shaking to increase the efficiency of extraction. The mixture was then filtered, and the filtrate was evaporated using a rotary evaporator set at 40°C to remove excess ethanol solvent. The resulting content was then freeze-dried to produce a light brown powder that was stored in the refrigerator.

The acetonic extract was prepared by taking 500 mg of *C. dichogamus* root powder into an extraction jar, adding 2000 ml of acetone gradually, and then shaken vigorously until a uniform consistency was obtained. The mixture was stirred continuously using a magnetic stirrer for 72 hours and then filtered. The filtrate was evaporated using a rotary evaporator whose operating temperature was set at 40°C for 4 hours. The resulting content was then placed into an amber colored bottle, covered with an aluminum foil, and then placed on a hot sand bath to get a consistent powder. The acetonic extraction was to be repeated to give enough yield that was required for the study.

### 2.4. Antimicrobial Studies

#### 2.4.1. Test Microorganisms

A fungal microorganism and four bacterial strains given in Table 1 were obtained from the stock cultures from the bacteriology laboratory at the PHPT department.

#### 2.4.2. Preparation of Cultures

The stock cultures were prepared according to the Clinical Laboratory Standards Institute (CLSI). A loopful of the pure cultures of each microbe was suspended in 10 ml sterile physiological saline to give a concentration equal to that of 0.5 MacFarland standards. According to Suffredini et al. [20], Gram-negative bacteria are never susceptible to plant extracts at a concentration lower than 200 mg/ml; thus, 250 mg/ml was a convenient dose for both Gram-positive and Gram-negative bacteria without the risk of nonspecific interaction. Stock solutions of 250 mg/ml were prepared by dissolving 1 g of the plant extracts (acetonic, aqueous, and hydroethanolic) in 1 ml of 1% dimethyl sulfoxide (DMSO); then, 3 ml of sterile molten Mueller Hilton was added to make 4 ml. Susceptibility studies were conducted according to the protocol described by Debalke et al. [21] with minor modifications. As described in the protocol [21], two-fold serial dilutions of 125 mg/ml, 62.5 mg/ml, 31.25 mg/ml, 15.63 mg/ml, and 7.8 mg/ml were made from the stock solution. The various dilutions made it easier to point out the exact concentration at which there was a complete bacterial inhibition. 1% DMSO was used as negative control while cephalexin, a broad-spectrum antibiotic, was used as a positive control for both Gram-positive and Gram-negative bacteria. Fluconazole was used as a positive control for the fungal microorganism.

#### 2.4.3. Microbroth Dilution Technique

The microbroth method as described by Muia et al. [22] was used, where five culture tubes containing 2 ml sterile Mueller Hilton Broth were arranged. Two-fold serial dilutions were made from the stock solution. Using a micropipette, 0.1 ml of each microorganism was inoculated into every tube of diluted plant extract. The bacterial organisms were then incubated at 37°C for 24 hours, while the fungal organism was placed at room temperature for 24 hours. The observed lowest concentration of the plant extracts that retained its inhibitory effect resulting in no visible growth (absence of turbidity) of microorganism was recorded as the minimum inhibitory concentration (MIC) value of the extract. For the determination of MBC, all tubes that showed no visible bacterial growth were aseptically cultured in sterile molten agar using the pour plate method and incubated. The lowest concentration of the plant extract that shows no visible bacterial growth was noted as the MBC value.

#### 2.4.4. Agar Well Diffusion Method

The agar well diffusion method was carried out as described by Clinical Laboratory Standards Institute (CLSI). Each test microorganism was spread on aseptically prepared nutrient agar by the use of a swab. Holes of 7 mm in diameter and 8 mm in depth were made using a sterile cork borer. 0.1 ml of the test extracts of varying concentrations were placed into the wells and allowed to stand on the bench for an hour for proper disperse into agar and incubated for 24 hours. The diameter of the zone of inhibition was measured in millimeters.

### 2.5. Brine Shrimp Lethality Studies

#### 2.5.1. Hatching of the Brine Shrimp Eggs

Brine shrimp eggs were hatched according to the method described by Nguta et al. [23], where the brine shrimp eggs were incubated and hatched in a shallow rectangular plastic container containing marine salt solution which was made by dissolving 33 g of marine salt in a liter of distilled water. The plastic container had unequal chambers separated by a wall with 2 mm holes. Approximately 50 mg of viable brine shrimp eggs was sprinkled on the bigger chamber which was dark, while the smaller chamber was illumined by a 40 watts electric bulb. About 6 mg of dry yeast was sprinkled on the eggs to serve as food for the nauplii. Once hatched, the phototropic larvae swam from the dark chamber to the illuminated chamber leaving their egg shells behind. The hatching period was 48 hours [17, 19].

#### 2.5.2. Cytotoxicity Bioassay

The stock solutions of aqueous, acetone 50% ethanol extracts, and vincristine sulphate (positive control) were prepared by taking 0.1 g of each sample and dissolving it in 1 ml DMSO and then topped up to the 10 ml mark with marine salt solution [19]. The concentration of the stock solution was 10,000 *μ*g/ml. Dimethyl sulfoxide was used as a negative control, while vincristine sulphate was used as a positive control in the cytotoxicity bioassay. Ten nauplii (*Artemia salina*) were then transferred into graduated test tubes using a disposable pipette. Aliquots of 500 *μ*l, 50 *μ*l, and 5 *μ*l representing the three concentrations of 1000 *μ*g/ml, 100 *μ*g/ml, and 10 *μ*g/ml, respectively, of each sample were transferred into test vials. Five graduated tubes were set for each dose level per sample. Marine salt solution was added to all the test vials to make 5 ml volume. The tubes were kept at room temperature for 24 hours; then, the number of dead larvae was counted using a magnifying glass. The percentage mortality was determined for each dose level and controls. The median lethal concentrations (LC_50_) were determined from the dead counts within 24 hours using probit regression analysis.

### 2.6. Phytochemical Studies

The crude root extracts of *C. dichogamus* were qualitatively screened for the presence of flavonoids, alkaloids, saponins, phenols, tannins, terpenoids, cyanogenetic glycosides, anthraquinones, polyuronides, and mucilage according to the procedures described by Visweswari et al. [24], Usman et al. [25], and Trease and Evans [26].

### 2.7. Statistical Analysis

All experiments were performed in triplicates; data were entered into Statistical Package for Social Sciences (SPSS), version 23, and the results are provided as mean ± SEM. One-way analysis of variance (ANOVA) and posthoc ANOVA using the Tukey HSD test was used to compare the differences in means among and between groups, respectively. Differences (among and between groups) were considered to be statistically significant at *p* < 0.05.

## 3. Results

### 3.1. Percentage Yield of Plant Material

The aqueous extract gave a percentage yield of 6.05% w/w, hydroethanolic extract gave 24.50% w/w, and acetone extract gave 1.29% w/w. The highest percentage yield was obtained from the aqueous extract, while the lowest yield was from the acetone extract.

### 3.2. Antimicrobial Activity

A varying antimicrobial activity was shown by the three extracts when investigated against the five microorganisms (Tables 2 and 3).

All the extracts were active against Gram-positive bacteria, *B. cereus* and *S. aureus*, while they were totally inactive against Gram-negative bacterial strains of *P. aeruginosa* and *E. coli* at a concentration of 250 mg/ml. The extracts were also active against the fungal organism, *Candida albicans.* Among the Gram-positive bacteria, the acetone extract of *C. dichogamus* exhibited the highest zone of inhibition on *B. cereus* (17.33 ± 0.58) at 250 mg/ml (Table 2) with an MIC value of 10.42 mg/ml (Table 3).

### 3.3. Brine Shrimp Cytotoxicity Assay

**Figure 1:**
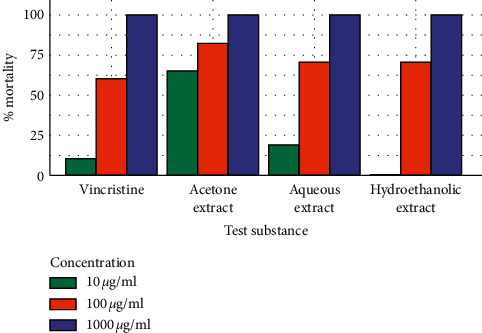
The comparison of the mortality induced by the crude root extracts (acetone, aqueous, and hydroethanolic) of *C. dichogamus* and vincristine sulphate.

The results of the toxicity of the various extracts against brine shrimp larvae are shown in Figure 1 and Table 4. Acetonic extract had the highest toxicity (LC_50_ 4.148 *μ*g/ml) followed by aqueous extract (LC_50_ 42.61 *μ*g/ml). The hydroethanolic extract displayed the least toxicity (LC_50_ 76.09 *μ*g/ml). All the extracts demonstrated LC_50_ values which were less than 100 *μ*g/ml. The results obtained have shown that the acetonic extract was more toxic than the control drug (vincristine sulphate), which had a LC_50_ value of 65.04 *μ*g/ml.

### 3.4. Phytochemical Composition

The phytoconstituents detected in the acetonic, hydroethanolic, and aqueous crude root extracts of *C. dichogamus* were flavonoids, saponins, phenols, terpenoids, anthracenes, and polyuronides, as given in Table 5.

According to the results, alkaloids were present in acetonic and hydroethanolic extracts but not in the aqueous extract. Tannins were present in aqueous and hydroethanolic extracts of *C. dichogamus* but were absent in the acetonic extract. Cyanogenetic glycosides were absent in all the three extracts.

## 4. Discussion

The propagation of drug resistance strains of microbes has posed a great challenge to global public health [1]. For this reason, the development of new therapeutic agents is critical in the future management of infectious diseases. Plants and their secondary metabolites have shown that they are a reliable resource of future antimicrobial agents as they have the ability to combat a wide range of human pathogens. The purpose of the current study was to investigate the antimicrobial activity and to qualitatively evaluate the phytoconstituents of the crude root extracts of *C. dichogamus*. The safety of the extracts was also determined in the brine shrimp cytotoxicity bioassay.

In this study, the highest yield was seen in the aqueous extract (6.05%) followed by hydroethanolic extract (4.9%). Acetonic extract gave a very low yield of 1.29% that necessitated a second extraction.

All the extracts of *C. dichogamus* showed activity against Gram-positive bacteria (*B. cereus* and *S. aureus*) and the fungal organism *C. albicans*, while all of the extracts showed no activity against Gram-negative bacteria (*P. aeruginosa* and *E.coli*). The selective activity of the extracts towards the bacteria strains could be due to the presence of an impermeable barrier of lipopolysaccharide on the outer membrane of Gram-negative bacteria that inhibit diffusion of active compounds [27]. On the other hand, Gram-positive bacteria freely allow the direct contact of active constituents with the phospholipid bilayer of the cell membrane leading to enhanced ion permeability [28]. The presence of the antibacterial property in *C. dichogamus* had also been confirmed in another study [15] that reported that the ethanolic root extract of *C. dichogamus* showed a weak antibacterial activity against two *Mycobacterium* species, namely, *Mycobacterium indicus pranii* and *Mycobacterium madagascariense indicus*, giving a minimum inhibitory concentration (MIC) value of 1.25 mg/ml.

The results of this study indicated that the MIC of the three extracts was quite weak (10.4–166.7 mg/ml) as compared to the MIC range of the commonly available antibiotics which is in a range of 0.015–0.107 mg/ml [29]. This study therefore indicated that all the extracts had a weaker antimicrobial activity even when compared to the standard drugs (cephalexin and fluconazole) at the same concentration of 250 mg/ml. It was established that there was no significant difference (*p* > 0.05) in the mean zones of inhibition of the acetone, aqueous, and hydroethanolic extracts. On the other hand, there was a significant difference (*p* < 0.05) between the mean zones of inhibition recorded for the three extracts and the positive controls (cephalexin and fluconazole) under various concentrations. The antimicrobial results of this study substantiate the traditional claim of the plant to treat ailments that are of bacterial origin like tuberculosis, pneumonia, and urinary tract infections [5, 30].

The brine shrimp bioassay is a rapid, reliable convenient, and inexpensive bench top procedure that determines the median lethal concentration values of plant extracts in a brine shrimp medium. The classification of toxicity in the brine shrimp bioassay described by Nguta et al. [23] and Meyer et al. [31] were used in the current study. In this classification, LC_50_ > 1000 *μ*g/ml were considered nontoxic, values of LC_50_ between 500 and 1000 *μ*g/ml were considered weakly toxic, values of LC_50_ between 100 and 500 *μ*g/ml were considered moderately toxic and values of LC_50_ between 0 and 100 *μ*g/ml were considered to be strongly toxic. The results in the current study indicated that all the extracts were highly cytotoxic with acetonic extract (LC_50_ of 4.148 *μ*g/ml) being more toxic than aqueous (LC_50_ of 42.61 *μ*g/ml) and hydroethanolic (LC_50_ of 76.09 *μ*g/ml) extracts. The difference in the cytotoxicity in the three extracts could be attributed to the phytochemical ratios of tannins, alkaloids, flavonoids, saponins, phenols, and terpenoids in them [23]. The brine shrimp lethality assay is normally used to predict the presence of the cytotoxic activity against cancer cells below 100 *μ*g/ml; therefore, all the three extracts of *C. dichogamus* were potentially cytotoxic with LC 50 values below 100 *μ*g/ml.

The cytotoxic agents work by interrupting the growth of cells at particular levels, especially those cells that exhibit a rapid growth. Their main mechanism of action may be due to arrest of cell cycle, induction of apoptosis, or inhibition of angiogenesis. In the current study, the overlap in confidence intervals of vincristine sulphate (65.04 (46.07–92.17)) and those of aqueous (42.61 (28.86–62.26)) and hydroethanolic extracts (76.09 (58.69–133.33)) suggests that there is no significant difference (*p* > 0.05) in the lethality induced by vincristine sulphate and that of aqueous and hydroethanolic extracts of *C. dichogamus.* However, there is a significance difference (*p* < 0.05) in the lethality of the acetonic extract and the control drug, since the LC_50_ value of the acetonic extract was too low with no overlap with the control drug. The acetonic extract was therefore more lethal than vincristine sulphate. The difference in the cytotoxicity in the three extracts could be attributed to the phytochemical ratios of tannins, alkaloids, flavonoids, phenols, and terpenoids in them [23].

The cytotoxicity report of this study resonates with that perfirmed by Magadula [15] who reported that ethanolic root extract of *C. dichogamus* gave the LC_50_ value of 40.70 *μ*g/ml. The cytotoxic property of the root of *C. dichogamus* was confirmed by a study performed by Aldhaher et al. [17] who reported the presence of a cytotoxic compound (10-epi-Maninsgin D) in the root of *C. dichogamus* that was viable against CACO (human colorectal adenocarcinoma) cell line with significant inhibition of cellular proliferation. Another study performed later reported that a sesquiterpenoid known as furocrotinsulolide isolated from the methanolic root extract of *C. dichogamus* recorded a modest cytotoxic activity against cancer cells at 30 *μ*m when tested on CACO-2 cell line [32]. These compounds could be responsible for the recorded cytotoxicity in the brine shrimp assay seen in the current study.

The phytochemical analysis in the current study confirmed the presence of saponins, phenols, polyuronides, tannins, triterpenoids, anthracenes, and flavonoids in aqueous, acetonic, and hydroethanolic root extracts of *C. dichogamus.* The alkaloids were present in acetonic and hydroethanolic extracts but not in the aqueous extract. The cyanogenetic glycosides were absent in the aqueous, hydroethanolic, and acetonic extracts. Tannins were present in aqueous and hydroethanolic extracts of *C. dichogamus* but were absent in the acetonic extract.

The presence of phytochemical constituents seen in this study like phenols and saponins was confirmed by a study performed by Johns et al. [18], while the presence of more than 20 diterpenoids was reported by Aldhaher et al. [17]. The presence of tannins, phenols, saponins, and alkaloids in reported in this study is inconsistent with the report given by Magadula [15] who indicated that those components were absent in the ethanolic root extract of *C. dichogamus*. This difference could be because of the geographical difference of the plant material.

In the current study, tannins were not detected in the acetonic crude root extract. However, for the aqueous and hydroethanolic root extracts, the amounts present were undetectable using GC-MS. The presence of tannins was also reported in another study performed by Johns et al. [18] who indicated that the methanolic root extract of *C. dichogamus* had a tannin content of 5.1 mg/g dry weight. Tannins are strong antioxidants because of the free radicle scavenging property in them. The fair content of tannins has been found useful in the medical field because tannins have antitumor, antimicrobial, and antiseptic properties [28]. This could be the source of the antimicrobial property in the extracts of *C. dichogamus*.

Studies have revealed that important pharmacological properties such as antiproliferative [17], anti-inflammatory [33], and insecticidal [13] activities which were present in *C. dichogamus* could be attributed to the high concentration of terpenoids [34] whose presence was also confirmed in the current study. Another study [32] also indicated that approximately 25 terpenoids were isolated from the root extracts of *C. dichogamus*. The presence of terpenoids in the extracts of *C. dichogamus* explains its use in the management of respiratory ailments such as cough, asthma, and chest pains and as terpenoids act to soothe the irritated mucous membrane lining the respiratory tract. Triterpenoids also exhibit antibacterial activities making the plant useful in treating respiratory infections that are of bacterial origin [15]. Reports have also established that in the pharmaceutical industry, terpenoids such as triterpenoids, sesquiterpenoids, and diterpenoids are used as anthelmintics, insecticides, and antibiotics [28].

The presence of phenolic compounds reported in this study was also confirmed in another study [18]. These compounds could be the reason why the plant is used to alleviate inflammatory conditions along the respiratory tract like asthma, pneumonia, pharyngitis, tuberculosis, and common cold. This is because phenolic compounds have been shown to protect the body cells against oxidative damage that cause inflammation in body tissues [35]. Kaul et al. [36] and Khanam et al. [28] reported that flavonoids give a reduced incidence of upper respiratory tract infections (URTI) because of their physiological effects on humans including antibacterial, antiviral, antiallergy, anti-inflammatory, antioxidant, and anticancer [23, 29]. In addition, reports have indicated that flavonoids reduce inflammation by reducing the size of NF-kB and stopping the replication and proliferation of two notorious viral sources of URTIs [36]. There is a positive correlation between increased consumption of flavonoids and reduced risk of respiratory infections, cardiovascular illness, and cancer illness [23, 29]. This supported the traditional use of *C. dichogamus* in the management of respiratory infections. Alkaloids inhibit the cyclooxygenase pathways which in turn inhibit inflammatory cytokines and interleukins that cause pain. Studies have also shown that alkaloids possess bacterial, antimalarial, and antispasmodic properties [28]. The presence of all the reported phytoconstituents plays a role in the observed antimicrobial and cytotoxic properties. This is due to the fact that the occurrence and quantities of the secondary metabolites determine the bioactivity of the plant [37].

This study is important because it served as a starting point in the discovery of new cytotoxic agents and the unveiling of the potent phytoconstituents in *C. dichogamus*. It also confirms the traditional claim of the presence of the antimicrobial activity in *C. dichogamus* and forms a basis for dose regulation of traditional preparations of *C. dichogamus* to avoid undesirable toxic effects.

## 5. Conclusions

The results of the current study confirmed that *C. dichogamus* possess a moderate antimicrobial activity and is highly toxic. The study also demonstrated that the roots of *C. dichogamus* are a good source of beneficial phytoconstituents. Despite the low yield, the acetonic extract demonstrated the highest antibacterial and antifungal activities against the tested microorganisms. The high cytotoxicity of *C. dichogamus* will limit its use as an antimicrobial agent. The previous statistical analyses performed on the antimicrobial and cytotoxic activities on different extracts at various concentrations were corroborated with the present findings and supported the traditional use of the plant as an antimicrobial agent. Thus, further study is required for dose adjustment among the communities that use the plant for curative purpose. Studies to determine the mechanism of action of this plant as an antimicrobial agent and cytotoxic agent are needed. Moreover, research is needed to isolate and identify the active phytoconstituents responsible for the cytotoxic activity in *C. dichogamus* for development of future anticancer drugs.

## Figures and Tables

**Table 1:** Microbes used in the antimicrobial studies.

Name of microorganism	Microbe type	Gram stain	Strain type
*Bacillus cereus*	Bacteria	Positive	ATCC 11778
*Staphylococcus aureus*	Bacteria	Positive	ATCC 25923
*Escherichia coli*	Bacteria	Negative	ATCC 25922
*Pseudomonas aeruginosa*	Bacteria	Negative	ATCC27853
*Candida albicans*	Fungus	—	ATCC 102231

**Table 2:** Antimicrobial activity of *C. dichogamus* root extracts on various concentrations using the agar well diffusion technique.

Microorganisms	Concentration	Zone of inhibition (MM)				
	mg/ml	Acetone extract	Hydroethanolic extract	Aqueous extract	Positive control	Negative control
					Cephalexin	
*Bacillus cereus*	250	17.33 ± 0.33	14.33 ± 0.33	12.83 ± 0.93	29.67 ± 2.48	0.00 ± 0.00
	125	16.33 ± 0.88	12.33 ± 0.33	10.33 ± 0.33		
	62.5	13.67 ± 0.88	9.5 ± 1.04	8.50 ± 0.5		
	31.25	5.67 ± 2.85	5.33 ± 2.68	2.50 ± 2.50		
	15.63	0.00 ± 0.00	0.00 ± 0.00	0.00 ± 0.00		
	7.81	0.00 ± 0.00	0.00 ± 0.00	0.00 ± 0.00		

*Staphylococcus aureus*	250	12.33 ± 0.88	11.5 ± 0.29	10.67 ± 0.44	28.67 ± 0.58	0.00 ± 0.00
	125	11.33 ± 0.33	10.33 ± 0.33	9.33 ± 0.60		
	62.5	9.67 ± 0.33	8.17 ± 0.44	8.00 ± 0.00		
	31.25	2.67 ± 2.67	0.00 ± 0.00	2.50 ± 2.50		
	15.63	0.00 ± 0.00	0.00 ± 0.00	0.00 ± 0.00		
	7.81	0.00 ± 0.00	0.00 ± 0.00	0.00 ± 0.00		

*Pseudomonas aeruginosa*	250	0.00 ± 0.00	0.00 ± 0.00	0.00 ± 0.00	27.33 ± 0.58	0.00 ± 0.00

*Escherichia coli*	250	0.00 ± 0.00	0.00 ± 0.00	0.00 ± 0.00	28.66 ± 4.16	0.00 ± 0.00

					Fluconazole	
*Candida albicans*	250	15.00 ± 0.58	12.00 ± 0.58	9.33 ± 0.33	29.00 ± 2.52	0.00 ± 0.00
	125	13.67 ± 0.33	10.33 ± 0.33	8.83 ± 0.33		
	62.5	10.67 ± 0.33	8.33 ± 0.33	5.33 ± 2.67		
	31.25	5.33 ± 2.67	5.17 ± 2.59	2.50 ± 2.50		
	15.63	0.00 ± 0.00	0.00 ± 0.00	0.00 ± 0.00		
	7.81	0.00 ± 0.00	0.00 ± 0.00	0.00 ± 0.00		

Zones of inhibition were expressed as mean ± SEM of the triplicate experiments. 0.00 = no activity.

**Table 3:** Average MICs and MBCs for acetone, aqueous, and hydroethanolic crude extracts of *C. dichogamus* against the test microorganisms.

Test organism	Extracts					
	Acetone		Aqueous		Hydroethanolic	
	MIC	MBC/MFC	MIC	MBC/MFC	MIC	MBC/MFC
*B. cereus*	10.42	166.67	13.03	104.67	10.41	62.5
*S. aureus*	13.02	83.33	31.25	83.33	10.41	125
*P. aeruginosa*	>250	>250	>250	>250	>250	>250
*E. coli*	>250	>250	>250	>250	>250	>250
*C. albicans*	31.25	104.167	67.7133	83.33	83.33	166.67

MIC, minimum inhibitory concentration; MBC, minimum bactericidal concentration; MFC, minimum fungicidal concentration.

**Table 4:** The toxicity profile of the acetonic, aqueous, and hydroethanolic root extracts of *C. dichogamus* as compared to vincristine sulphate.

Sample	Average mortality per test dose			Lethal concentration	Toxicity
	10 *μ*g/mL	100 *μ*g/mL	1000 *μ*g/mL	LC_50_ (95% confidence interval)	Meyer's criteria
Vincristine sulphate	3	31	50	65.04 (46.07–92.17)	Highly cytotoxic
Acetone extract of *C. dichogamus*	33	43	50	4.148 (0.58–9.87)	Highly cytotoxic
Aqueous extract of *C. dichogamus*	8	35	50	42.61 (28.86–62.26)	Highly cytotoxic
Hydroethanolic extract of *C. dichogamus*	0	35	50	76.09 (58.69–133.33)	Highly cytotoxic

**Table 5:** Phytochemical analysis of the aqueous, hydroethanolic and acetonic extracts of *Croton dichogamus*.

Test	Aqueous extract	Hydroethanolic extract	Acetonic extract
Flavonoids	+	+	+
Alkaloids	−	+	+
Saponins	+	+	+
Phenols	+	+	+
Tannins	+	+	−
Terpenoids	+	+	+
Mucilage	+	+	+
Cyanogenetic glycosides	−	−	−
Anthraquinones	+	+	+
Polyuronides	+	+	+

Key (+) means presence of phytochemical and (−) means absence of phytochemical means.

## Data Availability

The data used to support the findings of this study are available from the corresponding author upon request.
